# Harnessing sunflower stalk-based bowl for sustainable tobacco seedling and cultivation: influence on rhizosphere microbiome and carbon cycling

**DOI:** 10.3389/fmicb.2025.1661023

**Published:** 2025-10-09

**Authors:** Xiaolong Shi, Menglin Ge, Suting Hou, Qing Han, Anning Xu, Chunji Jiang, Xin Ma

**Affiliations:** ^1^College of Agronomy, Shenyang Agricultural University, Shenyang, China; ^2^China National Tobacco Corporation Liaoning Provincial Company, Shenyang, China

**Keywords:** tobacco, sunflower stalk valorization, seedling cultivation, rhizoshere microbiome, microbial ecological function

## Abstract

Microbes in the rhizosphere make significant contributions to nutrient cycling and plant health maintenance. Tobacco is an important commercial crop, and the methods used for seedling cultivation significantly influence the rhizosphere soil microenvironment. Compared to conventional seedling practices, the use of agricultural waste—such as sunflower straw—to fabricate biodegradable nursery containers represents an environmentally friendly alternative technology. This approach may exert specific potential effects on the structure and function of microbial communities in the tobacco rhizosphere. In this study, Yunyan-301 was used as the test material, and field experiments were carried out at two test sites (BT and HCZ). Each site included two treatments: conventional seedling cultivation (CK) and sunflower straw-based natural bowl seedling cultivation (T). While tobacco was harvested, a rhizosphere soil sample of tobacco was collected for microbial analysis. The results showed that sunflower straw-based natural bowl seedling cultivation led to a less diverse but functionally specialized microbial community. The specific alterations in the abundance of core taxa (e.g., Proteobacteria, Acidobacteria, *Sphingomonas*, *Pseudoduganella*, *Luteitalea*) suggest a potential ecological advantage, where the enriched community may be more efficient in utilizing sunflower straw-derived compounds and providing host-beneficial functions. Predictions of microbial community functions revealed that sunflower straw-based natural bowl seedling cultivation significantly enhanced the capacity of carbon fixation and oxidative phosphorylation, effectively improving the metabolic activity and carbon cycling ability of tobacco rhizosphere microbes. In summary, sunflower straw-based natural bowl seedling cultivation effectively alter the microenvironment of tobacco rhizosphere soil, thereby enriching functional microbial taxa and related metabolic pathways that were beneficial to soil health and tobacco growth. However, its effect was modulated by the environmental background of the test sites. In future research and actual production, further optimization of the sunflower straw-based natural bowl seedling cultivation should be conducted, and attempts should be made to assist in green and sustainable tobacco cultivation from the perspective of microbial community management.

## 1 Introduction

Tobacco (*Nicotiana tabacum* L.) is a crucial economic crop that plays a pivotal role in the national economy (Chen et al., 2022). As the world’s largest producer and consumer of tobacco, China’s tobacco industry contributes substantial tax revenues and provides employment for millions of farmers, serving as a vital pillar for rural revitalization and regional economic development. However, the sustainability of tobacco production is challenged by constraints in seedling cultivation. Conventional methods are plagued by issues such as soil-borne diseases, prolonged seedling periods, and low transplant survival rates, which are often linked to detrimental changes in soil microecology and ultimately hinder the industry’s sustainable development (Antoneli et al., 2023). In this context, innovative seedling cultivation techniques have emerged as critical breakthroughs for optimizing tobacco cultivation systems and achieving green sustainable development. Among these, utilizing agricultural waste (e.g., sunflower straw) to produce biodegradable nursery pots has shown promise not only in improving seedling quality but also in modulating the rhizosphere microenvironment. Yet, how this environment-friendly alternative specifically influences the structure and function of rhizosphere microbial communities—a key determinant of plant health—remains poorly understood.

In production, commonly used methods for tobacco seedling nursery include seedbed seedling technology and floating-seedling technology. Among emerging innovations, the seedling bowl technique has attracted considerable attention due to its efficient use of space, improved seedling uniformity, and enhanced root protection, which collectively contribute to higher transplant survival rates. While traditional plastic bowls offer mechanical strength and effective root containment, their widespread use poses environmental risks such as microplastic pollution from improperly discarded residues—a growing concern in sustainable agriculture (Piehl et al., 2018; Qi et al., 2018). In response, research on biodegradable alternatives derived from agricultural waste has gained momentum (Anirudh et al., 2024; Qi et al., 2023). Sunflower straw, in particular, has emerged as a promising material for manufacturing eco-friendly seedling bowls. Its rigid structure provides mechanical protection during handling and transport, while its porous internal architecture, high cellulose content, structural stability conferred by lignin, and substantial potassium content make it ideally suited for supporting seedling growth. Using sunflower straw as a seedling bowl can effectively ensure the survival rate of tobacco seedlings, and transplanting to the field is more convenient. After transfer, the roots of tobacco seedlings can be well buffered and grown in the straw, avoiding the influence of unfavorable factors in the soil when the root system is not fully developed. Moreover, as the straw decomposes, it releases bioactive compounds such as phenolic acids and soluble carbon, which can stimulate microbial activity and modulate plant–soil feedback (Bailey and Lazarovits, 2003). This gradual degradation process also promotes a stable rhizosphere environment—an important factor since the assembly and stability of microbial communities require extended periods of development (Kaurin et al., 2020). Thus, sunflower straw-based bowls not only support early root growth but may also contribute to the stabilization of the rhizosphere microbial community, offering a synergistic benefit for sustainable tobacco production.

Rhizosphere microbes, which are considered to be part of the second genome of plants, play a fundamental role in plant growth and health (Edwards et al., 2015). Microorganisms from the rhizosphere interact with tobacco roots can promote the improvement of soil quality by mobilizing nutrients and transforming organic matter in soil; In addition, the specific root metabolites released by plant roots may recruit a specific rhizosphere microbiome and then enhances plant adaptation and resistance to adverse environments (Mokabel et al., 2022). Gu et al. (2020) isolated plant growth-promoting rhizobacterial *Pseudomonas mediterranea* from tobacco rhizosphere and found that they exhibited strong antagonistic effects against a series of plant pathogenic fungi and bacteria. It has been reported that about 35–40% of straw carbon can be converted into different types of organic carbon during straw decomposition, such as soluble organic carbon and microbial biomass carbon, which can be used as energy and nutrient sources by soil microbes, thereby changing the composition of soil microbial communities (Su et al., 2020). Xie et al. (2025) conducted a shotgun metagenomic sequencing analysis and found that the treatment of straw notably altered soil microbial functions in corn rhizosphere, especially in carbon cycling and nutrient metabolism. Furthermore, a study on rice seedling cultivation using straw as a growth substrate showed that the bacterial biomass associated with carbon and nitrogen cycling was significantly greater in the straw-based treatment compared to the control. This approach also markedly improved the growth performance of the rice seedlings (Wang et al., 2021). Together, these findings offer further evidence that the rational incorporation of straw into agricultural practices actively enhances the soil carbon pool.

Therefore, investigating the specific effects of sunflower stalks bowl on the structure and ecological functions of tobacco rhizosphere microbial communities during the seedling stage will provide a theoretical basis for developing environment-friendly seedling cultivation techniques. In this study, sunflower straw was processed for used as seedling bowl, and high-throughput sequencing practices was applied to reveal the potential impact of this technique on the modulation of tobacco rhizosphere bacterial communities. This study is expected to provide a reference for the innovation of tobacco green cultivation technology and the utilization of straw resources.

## 2 Materials and methods

### 2.1 Site description

The experiment was conducted from June to September 2024 in Beipiao City, Liaoning Province. Experimental fields were established at two tobacco-growing sites, Beita Township (BT) and Heichengzi Township (HCZ), located approximately 20 km apart. The soil physicochemical properties for each site are summarized in Table 1. The experimental sites located between 41°20′ to 42°30′N latitude and 120°16′ to 121°20′E longitude. This region experiences a mid-temperate monsoonal continental climate, with an average annual temperature of 8.6 °C and annual rainfall of 509 mm. The frost-free period extends approximately 153 days annually, while the mean annual sunshine duration reaches 2,983 h.

**Table 1:** Physicochemical properties of soil at the experiment site.

Experimental sites	Soil types	Organic matter (%)	Total nitrogen (g/kg)	Available phosphorus (mg/kg)	Available potassium (mg/kg)	pH
BT	Sandy loam	1.24	1.32	8.89	169.00	7.80
HCZ	Sandy loam	1.00	1.21	8.29	207.56	7.64

### 2.2 Experimental design

The tobacco variety used in this study was Yunyan-301, a widely cultivated cultivar in China. A randomized complete block design (RCBD) was employed at each experimental site separately, with each site treated as an environmental block. Within each site, the two treatments, conventional seedling cultivation (CK) and sunflower straw-based natural seedling bowl cultivation (T), were randomly assigned to plots. This resulted in four distinct treatment groups: BTCK, BTT, HCZCK, and HCZT. Each treatment replicated three times.

**Figure 1:**
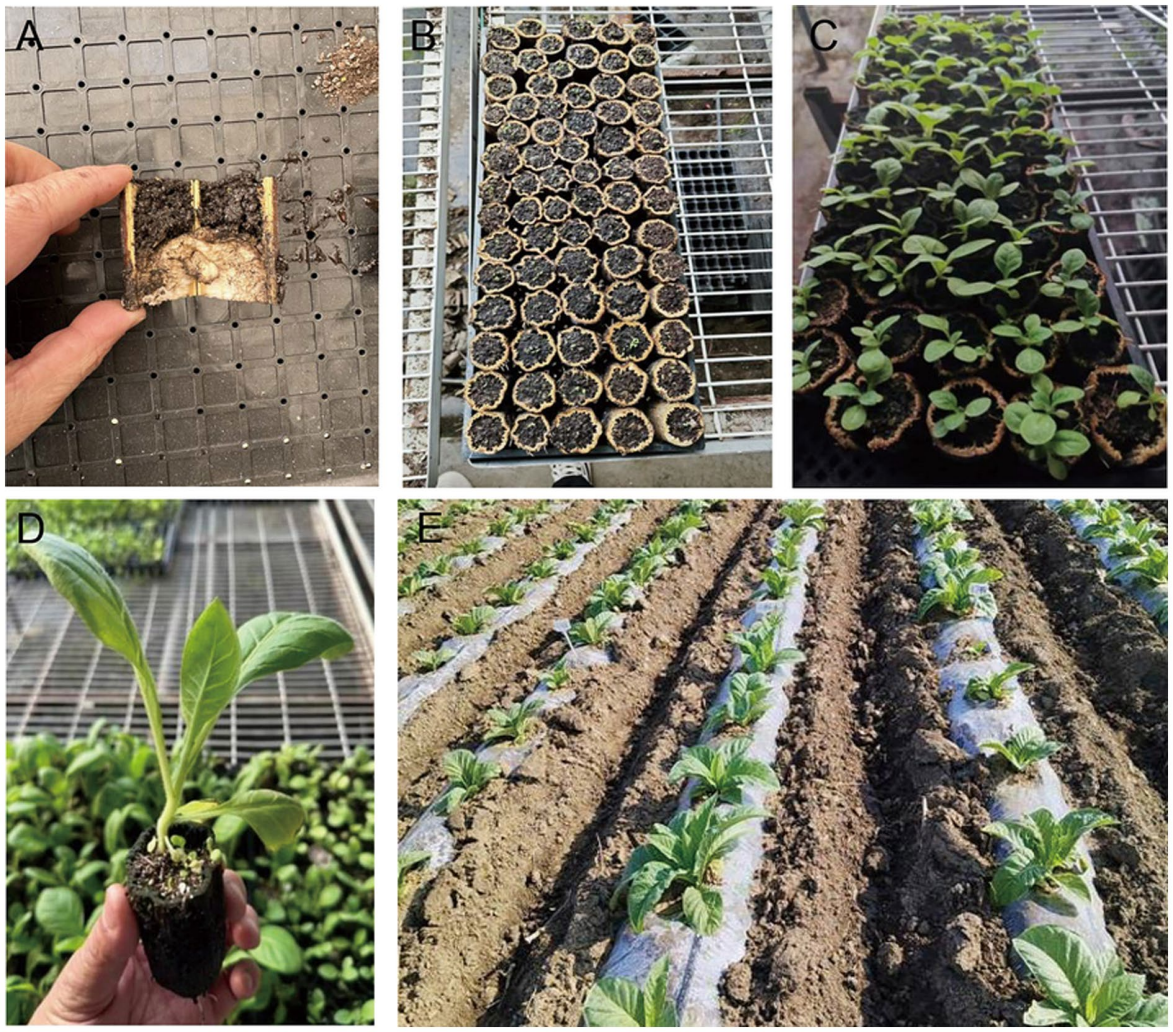
Flow chart of sunflower straw-based natural bowl seedling and transplanting. **(A)** The inner pulp of the sunflower straw was compacted; **(B)** Straw bowl substrate filling and seeding; **(C)** Tobacco seedling emergence; **(D)** Screening of tobacco seedlings before transplanting; **(E)** Field transplanting of tobacco seedlings.

For conventional seedling cultivation, 128-cell plastic trays were utilized with a substrate mixture of peat soil and vermiculite (3:1 ratio), followed by plastic film mulching for thermal insulation after sowing. The sunflower straw-based natural seedling bowl were prepared by sectioning uniform sunflower straw (4 cm diameter) into 5 cm segments, and the inner pulp of the sunflower straw were compacted to the bottom with a 3 cm diameter wooden rod. After filling the identical substrate, the tobacco seeds were evenly scattered on the surface of the sunflower straw-based natural seedling bowl, covered with a layer of thin soil, and watered to keep the soil moist (Figure 1).

Before transplanting, basal fertilizers were applied at a rate of 1,500 kg/ha decomposed farmyard manure combined with 50 kg/ha tobacco-specific compound fertilizer (N-P₂O₅-K₂O = 10–10-20). After 45 days of growing seedlings, uniform healthy tobacco seedlings with 4–5 leaves stage were transplanted into the field at a spacing of 1.1–1.2 m between rows and 0.45–0.5 m between plants, and a transplanting density was of 16,500–19,500 plants per hectare. All the tobacco plants were in-followed standard local field management throughout the whole growth periods.

### 2.3 Soil sample collection

Before the harvesting of tobacco leaves, five healthy plants were randomly collected in each plot. When taking soil samples, the topsoil was removed first, and then, the loose soil was shaken off from roots (the depth of roots was about 10 cm), and the soil closely attached to the root system was collected as rhizosphere soil by brushing it off. After sampling, the soil from five plants in each plot was mixed to create one composite sample, then sieved with a 2 mm mesh to remove large particles. Each composite sample were packed into three 15 mL sterilized centrifuge tubes (three biological triplicates), placed in iceboxes, and brought back to the laboratory, where they were maintained in the refrigerator at −80 °C for extracting soil DNA and high-throughput sequencing.

### 2.4 DNA extractions and microbial community sequencing

Fresh soil samples (0.5 g) were used for DNA extraction with the E.Z.N.A.® Soil DNA Kit (*MO BIO Laboratories* Inc., Carlsbad, CA, USA). The DNA extract was checked on 1% agarose gel, and the concentration and purity of DNA were determined by NanoDrop 2000 UV–Vis spectrophotometer (Thermo Fisher Scientific, Wilmington, NC, United States). The V1-V9 regions of the bacterial 16 s rRNA genes were amplified using primers 27F (5′- AGAGTTTGATCMTGGCTCAG-3′) and 1492R (5′-CRGYTACCTTGTTACGACTT-3′). The PCR amplification reaction system containing 5 × FastPfuBuffer 4 μL, 2.5 mM dNTPs 2 μL, Forward Primer (5 μM) 0.8 μL, Reverse Primer (5 μM) 0.8 μL, FastPfu Polymerase 0.4 μL, BSA 0.2 μL, Template DNA 10 ng, and finally DDH_2_O reached 20 μL. PCR reaction parameters were pre-denaturation for 5 min at 94 °C, followed by 40 cycles of 94 °C for 30 s, 55 °C for 30 s and 72 °C for 1 min, and held at 10 °C until analysis.

The PCR product was extracted from 2% agarose gel and purified using the AxyPrep DNA Gel Extraction Kit (Axygen Biosciences, Union City, CA, USA), according to manufacturer’s instructions, and quantified using Quantus™ Fluorometer (Promega, USA). High quality genomic DNA was used to prepare a SMRTbell library by using a SMRTbell Template Prep Kit 2.0 and then sequenced using the Sequel II sequencing platform (Shanghai Biozeron Biotechnology Co. Ltd., Shanghai, China).

### 2.5 Amplicon sequencing data processing

After sequencing data were generated, *PacBio SMRT* sequencing data were filtered for quality control (minimum number of passes = 3, minimum predicted accuracy = 0.99) employing SMRT Link (version 11.0) software, to obtain circular consensus sequence (CCS) reads for subsequent analysis. The SMRT portal assembly software was used to filter out the low-quality reads (<1,000 bp or >1800 bp), and further filter out barcode and primer sequences with lima pipeline to obtain high-quality CCS reads.

High quality sequence clustering was performed using UPARSE v10 software to obtain Operational Taxonomic Units (OTUs) based on 98.65% similarity (Edgar, 2013). The phylogenetic affiliation of each 16S rRNA gene sequence was analyzed by uclust algorithm^1^ against the Silva (SSU138.1) 16S rRNA database^2^ using confidence threshold of 80% (Quast et al., 2013). The community composition of each sample was subsequently determined at the phylum, class, order, family, genus, and species levels.

### 2.6 Bioinformatic and statistical analysis

Alpha diversity indices (Observed species, ACE, and Chao1) were calculated on a rarefied OTU table to correct for uneven sequencing depth. The rarefaction depth was set to 17,500 sequences per sample. Differences in alpha diversity between groups were statistically evaluated using the non-parametric Kruskal-Wallis test, followed by pairwise Wilcoxon rank-sum tests with Benjamini-Hochberg false discovery rate (FDR) correction. Beta diversity was assessed based on Bray-Curtis and Jaccard distances, the resulting distance matrices were visualized using Principal Coordinate Analysis (PCoA). Permutational Multivariate Analysis of Variance (PERMANOVA) with 9,999 permutations was applied using the adonis2 function in the vegan package to test for significant differences in microbial community structure between samples.

To identify communities or species that had a significant differential effect on sample delineation, we used the non-parametric factorial Kruskal-Wallis sum-rank test method to detect characteristics with significant abundance differences and to identify taxa that differed significantly in abundance. Finally, linear discriminant analysis (LDA) effect sizes (LEfSe) was used for identifying differences in population abundance, and assessing the magnitude of the effect of each species abundance on the differences (Segata et al., 2011). Functional potential of the microbial communities was predicted from the normalized OTUs table using PICRUSt2 against the KEGG database.

## 3 Results

### 3.1 Alpha diversity of the microbial communities

**Figure 2:**
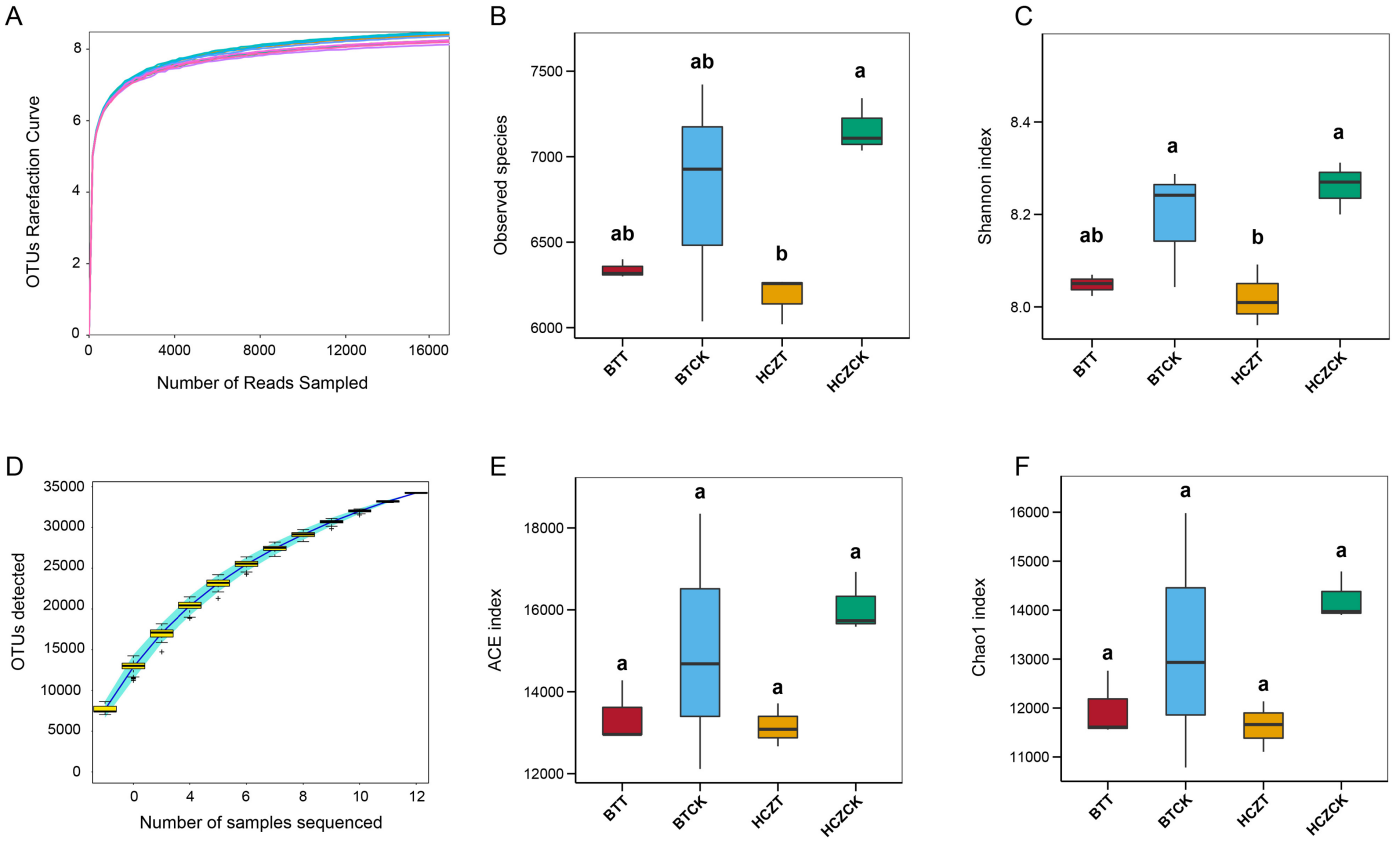
Alpha diversity of the microbial communities in different groups. **(A)** OTUs rarefaction curve. **(B)** Observed species. **(C)** Shannon index. **(D)** OTUs discovery curves. **(E)** ACE index. **(F)** Chao1 index. The lowercase letters indicate contrasts that are significantly different (*p* < 0.05) among different treatments.

As shown in Figure 2, the curve of OTUs numbers (Figure 2A) constructed with sequencing reads and the rate of new OTUs discovery curves (Figure 2D) gradually plateaued with increasing sequencing depth, indicating that the sample size was sufficient and the sequencing depth met the requirements for subsequent analyses. Compared with the control (BTCK and HCZCK), the Observed species, Shannon, ACE and Chao1 of the microbial community in the rhizosphere soil of tobacco under sunflower straw-based natural bowl seedling (BTT, HCZT) exhibited a decreasing trend. Notably, in the HCZ experimental site, significant decreases in Observed species and Shannon indices were observed in tobacco rhizosphere soil under the HCZT treatment (Figures 2B,C,E,F).

### 3.2 Dissimilarities between microbial communities

**Figure 3:**
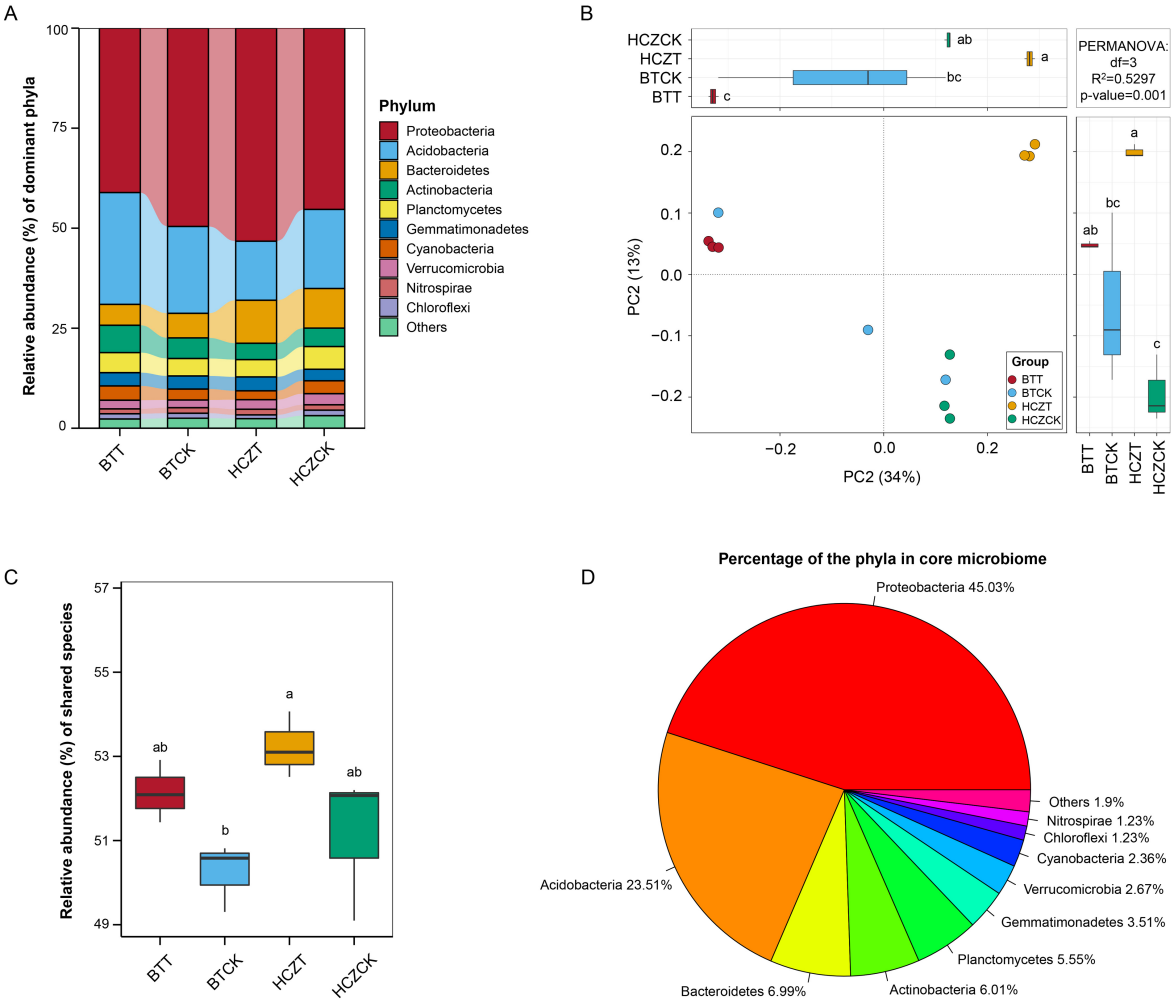
Microbial community differential analysis. **(A)** Percentage of microbial community abundance at phylum. **(B)** Permutational multivariate analysis of variance (PERMANOVA) of microbial communities of different groups at the phylum level. **(C)** Relative abundance of shared species. **(D)** Percentage of the phyla in core microbiome. The lowercase letters indicate contrasts that are significantly different (*p* < 0.05) among different treatments.

As shown in Figure 3A, the relative abundances of microbial phyla, including Proteobacteria, Acidobacteria, Bacteroidetes, Actinobacteria, Planctomycetes, and Gemmatimonadetes, were the highest among all soil samples. PERMANOVA analysis revealed that the variation in microbial community structure among samples had an R^2^ value of 0.5297 and a *p*-value of 0.001 (Figure 3B). Core microbiota identification analysis indicated that the total relative abundance of core OTUs in the rhizosphere soil samples of BTT and HCZT was higher than that in BTCK and HZCCK, although the differences were not statistically significant (Figure 3C). Among the identified core microbial taxa, Proteobacteria accounted for the highest proportion (45.03%), followed by Acidobacteria (23.51%), Bacteroidetes (6.99%), Actinobacteria (6.01%), and Planctomycetes (5.55%) (Figure 3D).

### 3.3 Dominant species among microbial communities

**Figure 4:**
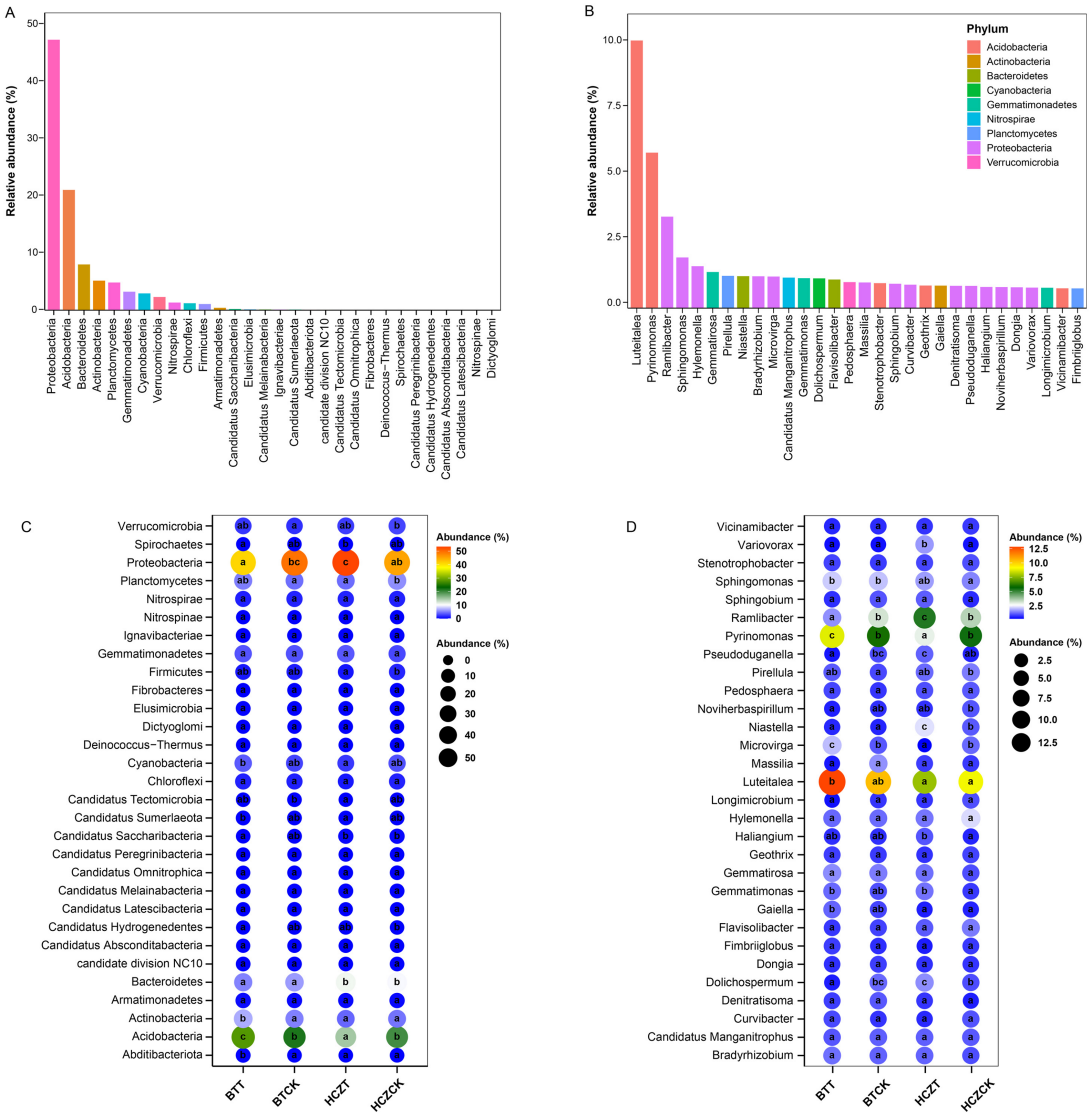
Dominant species among microbial communities. **(A)** Dominant phyla in microbial communities. **(B)** Dominant genera in microbial communities. **(C)** Relative abundance of dominant microbial phyla. **(D)** Relative abundance of dominant microbial genera. The lowercase letters indicate contrasts that are significantly different (*p* < 0.05) among different treatments.

Among the microbial phyla with the highest relative abundances (Figure 4A), the relative abundance of Proteobacteria was significantly higher in the rhizosphere soils of HCZT and BTCK compared to HCZCK and BTT, respectively. In the rhizosphere soils of BTCK and BTT, the relative abundance of Acidobacteria was higher than that in HCZCK and HCZT, whereas Bacteroidetes exhibited the opposite trend. Additionally, significant differences were observed in the relative abundances of Cyanobacteria, Actinobacteria, Spirochaetes, and Abditibacteriota between BTT and HCZT samples, but no significant differences were detected between BTCK and HCZCK samples (Figure 4C). At the genus level, *Luteitalea*, *Pyrinomonas*, *Ramlibacter*, *Sphingomonas*, and *Hylemonella* exhibited high dominance, with *Luteitalea* and *Pyrinomonas* belonging to the *Acidobacteria* phylum, and *Ramlibacter*, *Sphingomonas*, and *Hylemonella* classified under the Proteobacteria phylum (Figure 4B). The relative abundances of *Luteitalea* and *Pyrinomonas* were generally higher in the rhizosphere soils of BT compared to HCZ, whereas *Ramlibacter* showed the opposite pattern. Notably, the abundance differences of these three microbial genera between BTT and HCZT reached significant levels. Furthermore, significant differences were observed in the relative abundances of Var*iovorax*, *Pseudoduganella*, *Microvirga*, *Dolichospermum*, and *Gaiellad* between BTT and HCZT samples, but no significant differences were detected between BTCK and HCZCK samples (Figure 4D).

### 3.4 Identification of key microbial taxa

**Figure 5:**
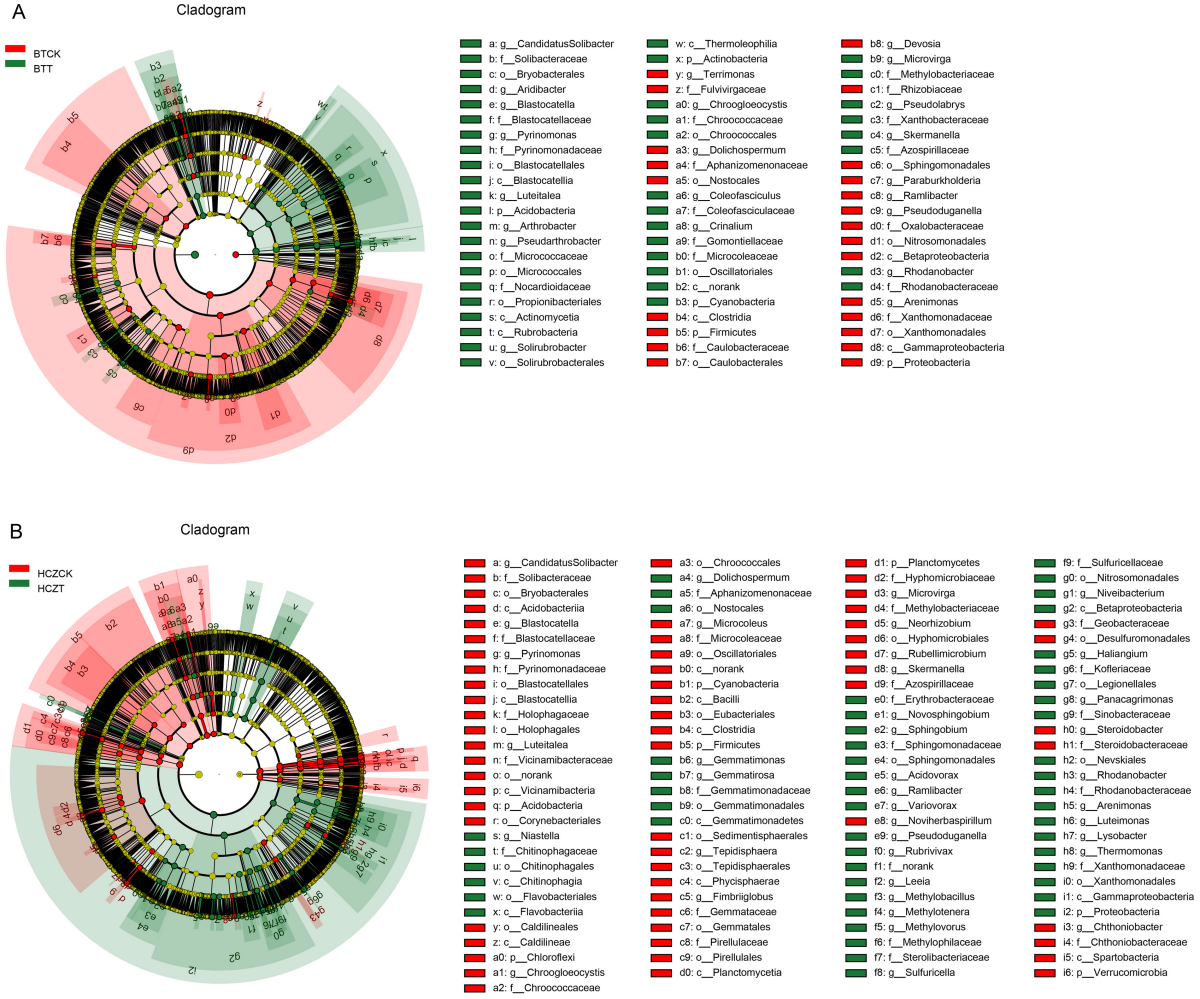
LEfSe analysis of the different microbial taxa in BTCK vs. BTT comparison group **(A)** and HCZCK vs. HCZT comparison group **(B)**.

Key microbial taxa were identified using LEfSe analysis and LDA scores (Supplementary Figure S1). In the BTCK vs. BTT comparison group, microbial phyla with higher LDA scores in the rhizosphere soil of BTCK included Proteobacteria, while the genus-level taxa were *Ramlibacter*, *Devosia*, and *Dolichospermum*. In the rhizosphere soil of BTT, microbial phyla with higher LDA scores were Acidobacteria and Actinobacteria, and the corresponding genus-level taxa were *Pyrinomonas*, *Luteitalea*, and *Microvirga* (Figure 5A). In the HCZCK vs. HCZT comparison group, microbial phyla with higher LDA scores in HCZCK rhizosphere soil included Acidobacteria, Planctomycetes, and Cyanobacteria, while the genus-level taxa were *Pyrinomonas*, *Luteitalea*, and *Microvirga*. In the rhizosphere soil of HCZT, microbial phyla with higher LDA scores were Proteobacteria, and the corresponding genus-level taxa were *Ramlibacter*, *Variovorax*, *Niastella*, and *Methylotenera*. Overall, the HCZCK vs. HCZT comparison group identified more differentially abundant microbial taxa (Figure 5B).

### 3.5 The functional predictions of microbial communities

**Figure 6:**
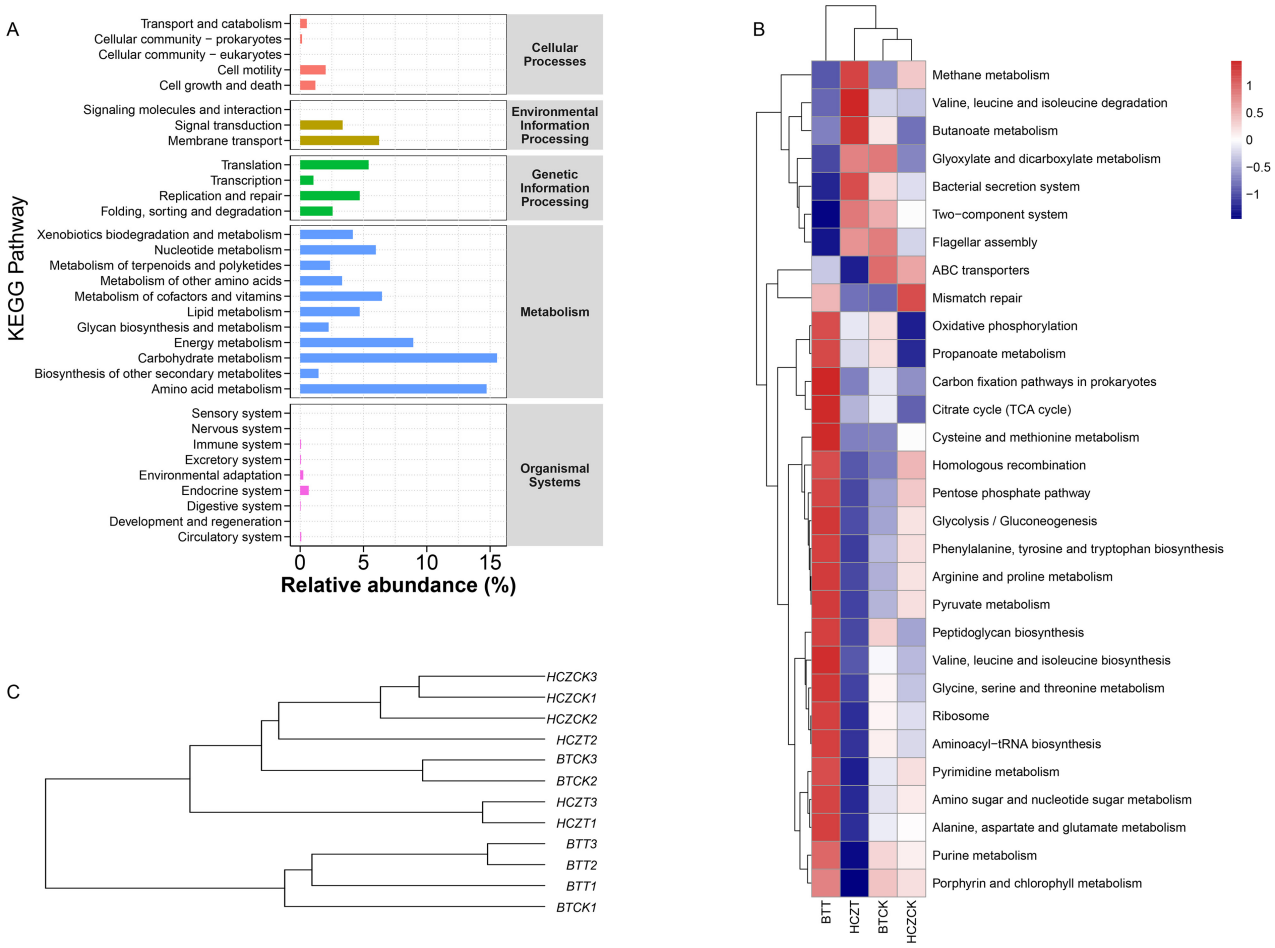
Functional prediction of microbial communities. **(A)** The bar graph shows the main potential function of the tobacco rhizosphere soil microbial community on KEGG level-1 and level-2. **(B)** Cluster heat map analysis on the relative abundance of KEGG microbial function at level-3 pathways between different groups. **(C)** The UPGMA clustering of the microbial function.

Functional prediction of the microbial community was conducted based on the KEGG database. The results showed that the “Metabolism” category accounted for the highest proportion in the level-1 KEGG pathway annotation. Further analysis at the level-2 revealed that “Carbohydrate metabolism,” “Amino acid metabolism,” “Energy metabolism,” and “Metabolism of cofactors and vitamins” exhibited significantly higher relative abundances compared to other functional categories (Figure 6A). At the level-3, key metabolic functions such as “Oxidative phosphorylation,” “Propanoate metabolism,” “Carbon fixation pathways in prokaryotes,” and “Citrate cycle (TCA cycle)” displayed higher relative abundances in the BTT and HCZTT samples compared to BTCK and HCZCK samples (Figure 6B). UPGMA clustering analysis indicated that the microbial community functions were most similar between BTCK and HCZCK, which indicated that the straw-based seedling cultivation treatment strongly influenced the functional potential of soil microbial communities in the tobacco rhizosphere (Figure 6C).

## 4 Discussion

Tobacco, as a critical economic crop, the dynamic changes of soil microbial community during its cultivation process exhibits profound impacts on tobacco plant health, nutrient absorption and disease prevention and control. In recent years, with the popularization of sustainable agriculture concepts, eco-friendly cultivation technologies utilizing agricultural waste (such as sunflower straw) as seedling substrates have garnered significant attention. This approach not only mitigates environmental pollution caused by conventional plastic seedling bowl but also potentially regulates the composition and function of microbial communities by altering the physicochemical properties of rhizosphere soil.

### 4.1 Effects of sunflower straw-based bowl seedling on the microbial communities of tobacco

Using sunflower straw-based natural seedling bowl ensures a longer development buffer period for the root system after tobacco transplantation. Additionally, the decomposition process of sunflower straw in the soil can alter the microenvironment of tobacco rhizosphere soil (such as soil C/N, pH, etc.). It reported that during the process of straw decomposition, approximately 40% of the carbon (C) in the straw can be converted into dissolved organic C and microbial biomass C, which can serve as energy and nutrient sources for soil microorganisms, thereby changing the composition of the soil microbial community (Su et al., 2020). Changes in the structural characteristics of the microbial community in the rhizosphere soil of tobacco play a significant role in the expression of its ecological functions. Many studies have found that the addition straw can provide more complex soil microenvironment for microorganisms, thereby increasing the diversity index of soil bacterial community, particularly under high nitrogen, or continuous straw return (Zhang et al., 2024). However, our study found that the diversity index of microbial community structure in tobacco rhizosphere soil did not show an increasing trend under the sunflower straw-based natural bowl seedling mode, but rather a significant decrease. The lack of significant response in microbial diversity to low amounts of sunflower straw may be attributed to microbial niche saturation, where the limited supplemental resources were insufficient to alter the established community structure or overcome community filtering mechanisms in the stable soil environment (Lu et al., 2017; Hargreaves et al., 2015). Additionally, it is also possible that the addition of crop straw can specifically recruit certain key microbial taxa, thereby influencing microbial community diversity (Guo et al., 2015). This selection is likely driven by the phenolic acids and complex lignin prevalent in sunflower straw. These compounds may serve as a selective substrate, stimulate microbial activity and modulate plant–soil feedback (Bailey and Lazarovits, 2003).

PERMANOVA analysis reflects the differences in microbial communities between sunflower straw-based natural bowl seedling cultivation and conventional seedling cultivation, with changes often associated with the impact of straw on the rhizosphere microenvironment of tobacco. For instance, the abundant carbon and nitrogen sources in the organic substrates of straw can promote the multiplication of specific functional microbes (Zhao et al., 2016). Additionally, plant straw may release various specific secondary metabolites during decomposition, further influencing the dynamic balance of microbial communities (Liu et al., 2023). Notably, sunflower straw-based natural bowl seedling may indirectly affect microbial activity by altering soil water retention and aeration capacities. For example, organic matrices typically have high water-holding capacities, which may provide a more stable environment for aerobic microbes while reducing soil redox potential, thereby promoting the growth of anaerobic microbes. Such complex environmental changes could lead to significant differences in microbial community structures. Research has shown that under organic matter input, the relative abundances of Acidobacteria and Proteobacteria in the soil exhibit different trends (Shi et al., 2022). Our study also found that in two experimental sites, the relative abundances of Acidobacteria and Proteobacteria in tobacco rhizosphere soil under straw-based natural bowl seedling and conventional seedling exhibited different trends. Acidobacteria and Proteobacteria are considered microbial taxa with different nutritional strategies (r-strategy microbes and k-strategy microbes), and the differences in their abundances may reflect the specific effects of different soil fertility levels and different seedling treatments on the microbial community structure of tobacco rhizosphere soil.

### 4.2 Key microbial taxa in microbial communities and the potential functional role

After comparing the microbial community structure between sunflower straw-based natural seedling bowl cultivation and conventional seedling cultivation, the identification of key microbial taxa is of great significance for understanding the ecological functions of microbial communities. The differences in core microbial taxa may reflect their adaptability to environmental changes and their potential roles in soil nutrient cycling, plant growth promotion or pathogen inhibition. The results showed that many microbial taxa, such as *Pyrinomonas*, *Luteitalea*, *Microvirga* under BTT treatment and *Ramlibacter*, Var*iovorax*, *Niastella*, *Methylotenera* under HCZT treatment, were functionally related to organic matter degradation in soil ecosystems, and influence soil carbon and nitrogen transformation and cycling by releasing specific substances (Chen et al., 2020).

Functional predictive analysis can further reveal the impact of sunflower straw-based natural bowl seedling cultivation on the functional aspects of the tobacco rhizosphere soil ecosystem. Research findings indicate that at the level 1 KEGG functional annotation, categories such as “Carbohydrate metabolism,” “Amino acid metabolism,” and “Energy metabolism” exhibit relatively high abundance. Notably, under sunflower straw-based natural bowl seedling cultivation, the relative abundance of “Carbon fixation pathways” in the level 3 KEGG functional annotation was significantly higher compared to conventional seedling cultivation. This observed increase in carbon fixation-related functions aligns with the understanding that organic matter inputs, such as those derived from sunflower straw, can enhance microbial activity via the “rhizosphere priming effect.” As demonstrated by Insam and Markt (2016), this mechanism simultaneously stimulates both carbon fixation (anabolism) and organic matter decomposition (catabolism) among rhizosphere microorganisms, collectively promoting the transformation and mineralization of soil organic matter. The enhanced metabolic activity under sunflower straw treatment suggests that this cultivation practice may activate decomposer microorganisms, thereby accelerating the mineralization of organic matter (Deng et al., 2021). Such functional enhancements likely contribute to an increased supply of available carbon sources in the soil, which could in turn improve nutrient availability and support the growth of tobacco root systems.

“Oxidative phosphorylation” is a metabolic pathway that generates ATP by utilizing energy released from the oxidation of nutrients (Luo et al., 2017). Under sunflower straw-based natural bowl seedling cultivation, the increased relative abundance of this pathway suggests a potential enhancement of energy production within the tobacco rhizosphere soil microbial communities. This may provide more energy for microbial metabolism, which could facilitate straw decomposition and improve nutrient availability in the rhizosphere. However, it is important to note that these functional inferences are based on 16S rRNA gene sequencing and performed by using PICRUSt2, which may not fully capture the actual enzymatic activities or *in situ* metabolic rates. Thus, the link between gene abundance predictions and improved ATP generation should be interpreted as a plausible mechanistic hypothesis rather than a confirmed outcome.

### 4.3 The ecological advantage and potential challenges of sunflower straw-based bowl seedling

From an ecological perspective, sunflower straw-based natural bowl seedling cultivation may demonstrate advantages in multiple aspects. First, its use as an organic substrate can reduce environmental pollution caused by traditional plastic seedling bowl, while increasing soil organic matter content, improving soil structure, and enhancing soil water retention and aeration capabilities. Second, the abundant carbon source in sunflower straw may provide more energy sources for microbes, thereby promoting the functional diversity of microbial communities. Additionally, sunflower straw may contain specific secondary metabolites (such as lignin, flavonoid compounds, etc.), which may regulate microbial community composition and further influence soil ecological functions.

However, sunflower straw-based natural bowl seedling cultivation may also face some potential challenges. For instance, the decomposition process of straw may require a certain amount of time, which could affect the nutrient supply during the initial stage of seedling growth. Additionally, some organic substrates such as sunflower straw can release volatile organic compounds or other toxic substances during decomposition, which might negatively affect microbial community stability and plant health. Moreover, the physicochemical properties of sunflower straw, such as its carbon-to-nitrogen ratio and lignin content, can vary significantly depending on its geographic origin, which may in turn influence the consistency and effectiveness of the method across different regions.

Given these potential variabilities and risks, long-term field studies are essential to evaluate the sustainability of this cultivation practice. Future research should focus on assessing the stability of the soil microbial community over time, as well as the long-term impacts on soil health under continuous application. Further investigations could also explore optimized straw amendment ratios, combinations with other organic amendments, and tailored agronomic practices to enhance the reliability and ecological benefits of this seedling cultivation system.

## 5 Conclusion

Overall, sunflower straw may influence the ecological functions of tobacco rhizosphere soil by altering the soil microenvironment and microbial community composition. Our results indicate that sunflower straw-based natural bowl seedling cultivation may promote the proliferation of certain beneficial microorganisms, including members of Proteobacteria, Acidobacteria, *Sphingomonas*, and *Luteitalea*. Additionally, functional prediction analysis suggests that this cultivation method may enhance carbon cycling-related functions, potentially improving soil nutrient supply capacity. Furthermore, it may strengthen the ability of tobacco rhizosphere microorganisms to participate in straw decomposition and nutrient cycling by increasing microbial energy metabolic processes. However, these functional insights are based on predictive bioinformatics methods and do not directly measure metabolic activity or enzyme expression, thus introducing inherent uncertainties. Future studies should therefore employ metagenomic or enzymatic assays to experimentally validate the predicted microbial functions and pathways.

## Data Availability

The datasets presented in this study can be found in online repositories. The names of the repository/repositories and accession number(s) can be found at: https://www.ncbi.nlm.nih.gov/, PRJNA1281805.

## Footnotes

^1^ https://github.com/topics/uclust

^2^ http://www.arb-silva.de
