# Supplementary figures and images for: Harnessing sunflower stalk-based bowl for sustainable tobacco seedling and cultivation: influence on rhizosphere microbiome and carbon cycling
Source: Front Microbiol. 2025 Oct 9;16:1661023. doi: 10.3389/fmicb.2025.1661023 (PMC12546116; doi:10.3389/fmicb.2025.1661023)

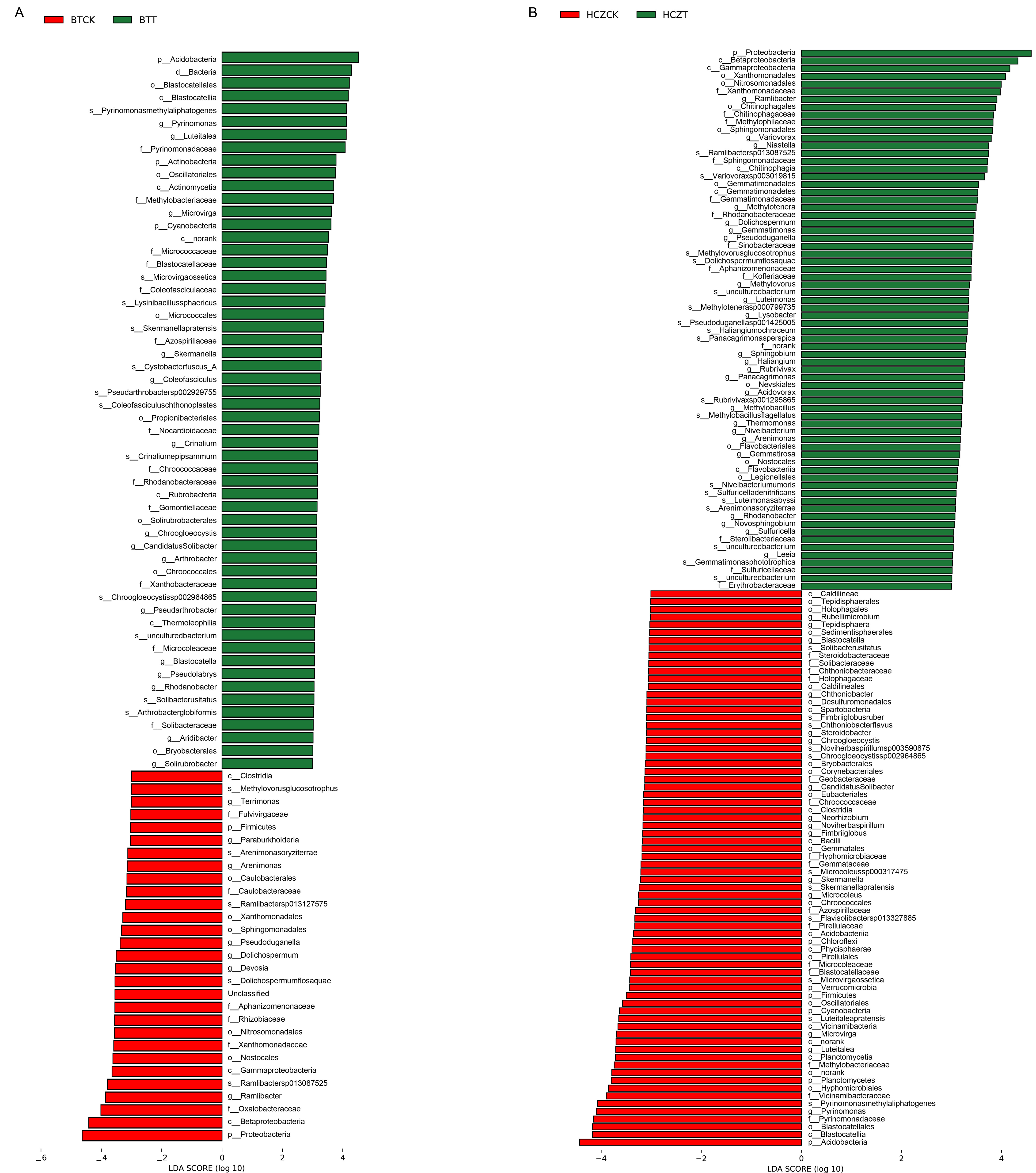

Supplement: Supplementary file 1 [file Image_1.jpeg]
